# Correction: Enhanced electrocaloric effect, energy storage density and pyroelectric response from a domain-engineered lead-free BaTi_0.91_Sn_0.08_Zr_0.01_O_3_ ferroelectric ceramic

**DOI:** 10.1039/d3ra90092d

**Published:** 2023-09-25

**Authors:** Hend Kacem, Ah. Dhahri, F. Aouaini, Z. Sassi, L. Seveyrat, L. Lebrun, J. Dhahri

**Affiliations:** a Université de Monastir, Faculté des Sciences de Monastir, Laboratoire de la Matière Condensée et des Nanosciences LR11ES40, 5000 Monastir Tunisia kacem.hend@gmail.com +216-92188163; b Université de Lyon, INSA-LYON, LGEF, EA682 F-69621 Villeurbanne France; c Department of Physics, College of Science and Humanities – Dawadmi, Shaqra University Riyadh Saudi Arabia; d Université de Sfax, Faculté des Sciences de Sfax, Laboratoire de Physique Appliqué B. P. 1171 Sfax Tunisia; e Department of Physics, College of Science, Princess Nourah bint Abdulrahman University P.O. Box 84428 Riyadh 11671 Saudi Arabia

## Abstract

Correction for ‘Enhanced electrocaloric effect, energy storage density and pyroelectric response from a domain-engineered lead-free BaTi_0.91_Sn_0.08_Zr_0.01_O_3_ ferroelectric ceramic’ by Hend Kacem *et al.*, *RSC Adv.*, 2022, **12**, 30771–30784, https://doi.org/10.1039/D2RA04914G.

The authors regret that two affiliations (affiliations *c* & *d*) were incorrectly shown in the original manuscript. The corrected list of affiliations is as shown here.

The Royal Society of Chemistry apologises for these errors and any consequent inconvenience to authors and readers.

## Supplementary Material

